# Prognostic Neurotransmitter Receptors Genes Are Associated with Immune Response, Inflammation and Cancer Hallmarks in Brain Tumors

**DOI:** 10.3390/cancers14102544

**Published:** 2022-05-21

**Authors:** Yuri Belotti, Serenella Tolomeo, Rongjun Yu, Wan-Teck Lim, Chwee Teck Lim

**Affiliations:** 1 Institute for Health Innovation and Technology, National University of Singapore, 14 Medical Drive, Singapore 117599, Singapore; yuri_belotti@bii.a-star.edu.sg; 2 Institute of High Performance Computing, Agency for Science, Technology and Research (A*STAR), 1 Fusionopolis Way, Singapore 138632, Singapore; serenella_tolomeo@ihpc.a-star.edu.sg; 3 Department of Pharmacology, Yong Loo Lin School of Medicine, National University of Singapore, 16 Medical Drive, Singapore 117600, Singapore; 4 Department of Management, Hong Kong Baptist University, 34 Renfrew Road, Hong Kong 999077, China; rongjunyu@hkbu.edu.hk; 5 Duke-NUS Medical School, 8 College Road, Singapore 169857, Singapore; darren.lim.w.t@singhealth.com.sg; 6 Division of Medical Oncology, National Cancer Centre Singapore, 11 Hospital Drive, Singapore 169610, Singapore; 7 Institute of Molecular and Cell Biology (IMCB), Agency for Science, Technology and Research (A*STAR), 61 Biopolis Drive, Singapore 138673, Singapore; 8 Department of Biomedical Engineering, National University of Singapore, 4 Engineering Drive 3, Singapore 117583, Singapore; 9 Mechanobiology Institute, National University of Singapore, 5A Engineering Drive 1, Singapore 117411, Singapore

**Keywords:** brain tumors, glioblastoma, TCGA, bioinformatics, transcriptomics, prognostic biomarker, precision medicine

## Abstract

**Simple Summary:**

Glioblastoma multiforme (GBM) is an aggressive form of glioma characterized by poor survival rates. The main cause of the limited efficacy of the current treatments and tumor recurrence is associated with the infiltration of GBM cells into the surrounding brain tissue. Until recently, peripheral nerves were believed to play a passive role in tumorigenesis; however, over the last decade, pioneering studies have highlighted their involvement in cancer initiation and progression by releasing neurotransmitters (NTs). In this study, we hypothesized that dysregulated genes encoding for neurotransmitter receptors (NTRs) could have a different association with patient survival in GBM and low-grade glioma (LGG). We identified 10 prognostic NTR genes that are progressively downregulated across cancer grades and are negatively correlated with genes associated with immune response, inflammation, and brain cancer hallmarks in LGG but not in GBM. We believe our findings shed new light on the role of neurotransmitters and their interactions with inflammation and immune response in the malignant progression of human gliomas.

**Abstract:**

Glioblastoma multiforme (GBM) is one of the most aggressive forms of cancer. Neurotransmitters (NTs) have recently been linked with the uncontrolled proliferation of cancer cells, but the role of NTs in the progression of human gliomas is still largely unexplored. Here, we investigate the genes encoding for neurotransmitter receptors (NTRs) by analyzing public transcriptomic data from GBM and LGG (low-grade glioma) samples. Our results showed that 50 out of the 98 tested NTR genes were dysregulated in brain cancer tissue. Next, we identified and validated NTR-associated prognostic gene signatures for both LGG and GBM. A subset of 10 NTR genes (*DRD1*, *HTR1E*, *HTR3B*, *GABRA1*, *GABRA4*, *GABRB2*, *GABRG2*, *GRIN1*, *GRM7*, and *ADRA1B*) predicted a positive prognosis in LGG and a negative prognosis in GBM. These genes were progressively downregulated across glioma grades and exhibited a strong negative correlation with genes associated with immune response, inflammasomes, and established cancer hallmarks genes in lower grade gliomas, suggesting a putative role in inhibiting cancer progression. This study might have implications for the development of novel therapeutics and preventive strategies that target regulatory networks associated with the link between the autonomic nervous system, cancer cells, and the tumor microenvironment.

## 1. Introduction

The most prevalent intracranial primary tumors in adults are gliomas, which originate from glial support cells [1,2]. In the World Health Organization (WHO) grade system, grade I–II gliomas are benign and are usually treated via surgical resection, whereas grade III–IV gliomas are malignant and significantly more difficult to treat [3]. Glioblastoma multiforme (GBM) is the most aggressive form of glioma, with more than 13,000 cases every year in the USA alone [4] and poor survival rates [4], highlighting the limitations of existing local and systemic therapies [5]. The main cause of the limited efficacy of the current treatments and subsequent tumor recurrence is associated with the infiltration of GBM cells into the surrounding brain tissue [6].

Until recently, the role of the peripheral nerves in tumorigenesis has been understudied, as they were believed to play only a passive role [7]. Over the last decade, a few pioneering studies have highlighted the involvement of nerves in cancer initiation and progression [8,9,10,11] (recently reviewed by Faulkner et al. [7] and Zahalka et al. [12]). Peripheral nerves are an essential component of the tumor microenvironment (TME) as they infiltrate every tissue and connect them to the central nervous system (CNS) [7]. They can stimulate cancer growth by releasing neurotransmitters (NTs) and neuropeptides, which are acting on receptors expressed by cancer cells [13,14]. For instance, adrenergic signals have been shown to stimulate β-adrenergic receptors expressed by tumor cells, and hence affect cancer development [15]. Specifically, adrenergic receptor activation elicits the secretion of the vascular endothelial growth factor (VEGF), angiogenesis, and enhances tumor progression [16,17]. Clinical studies have also shown that β-blocker intake is associated with prolonged survival in patients with prostate [18] and breast cancer [19], ovarian cancer [20], and multiple myeloma [21]. In addition, animal studies have shown that dopamine can prevent angiogenesis and that dopamine treatments suppress endothelial cell motility [22]. Furthermore, cancer cells are capable of eliciting the recruitment and development of new nerves in the tumor microenvironment (axonogenesis) via neurotrophic factor release and, in turn, the nerves release neurotransmitters that activate cancer growth and dissemination [7]. The current understanding of the interactions between cancer cells and NTs originates primarily from studies on cell lines and animal models (see Jiang et al. [23] for a detailed review).

The complex interactions between cancer cells, NTs, and other matrisomal components are increasingly recognized [24]. Various studies have provided evidence that dysregulated matrisomal components enable the prognostication and prediction of various malignancies [25,26,27,28,29,30]. Moreover, the TME plays a critical role in regulating cancer progression in brain cancers [2]. A putative link between these components is represented by inflammation [7,31,32]. Inflammation is known to drive many forms of cancer in humans [31]. Furthermore, inflammation-associated tumor development is elicited by a variety of immune cells [32] and cytoplasmic multimeric protein complexes, known as inflammasomes, appear to play a key role in orchestrating this mechanism [33,34,35]. Therefore, insights into the interactions between NTs, tumor cells, inflammation, and immune response may provide a more comprehensive picture of malignant progression in brain tumors. We hypothesized that the dysregulation of classical neurotransmitter receptors (NTRs) (i.e., dopamine, serotonin, gamma aminobutyric acid (GABA), glutamate, acetylcholine, epinephrine, and norepinephrine) could play a role in malignant progression. First, we aimed to identify whether genes encoding for NTRs were differentially expressed in brain cancer tissues when compared with matched normal tissue. We examined the gene expression data of low-grade glioma (LGG) and the glioblastoma multiforme (GBM) datasets acquired from the publicly available “The Cancer Genome Atlas (TCGA)” and the “Genotype-Tissue Expression (GTEx)” databases. Second, we sought to investigate the prognostic role of the identified differentially expressed genes for each brain cancer type and to identify the genes that were simultaneously prognostic for both cancer types and therefore potentially involved in the malignant progression from LGG to GBM. Next, we hypothesized the existence of potential correlations with recently identified genes associated with immune response, inflammasomes, and established cancer hallmarks [36]. Altogether, the overarching goal was to investigate the impact of neurotransmitter receptors in glioma progression to better understand their relationship with known cancer hallmarks and, specifically, with immune response and inflammation.

## 2. Materials and Methods

### 2.1. Differential Gene Expression Analysis

The HUGO Gene Nomenclature Committee (HGNC, www.genenames.org, accessed on 21 January 2021) was used to identify the genes encoding for classical neurotransmitter receptors. Then, a differential gene expression (DEG) analysis between tumor and normal tissue was conducted for each of the selected TCGA malignancies. The web-based tool GEPIA2 was used (www.gepia2.cancer-pku.cn, accessed on 21 January 2021). Specifically, the “Expression DIY” module was used. Each DE gene was inputted individually as “Gene” in GEPIA2, and all the TCGA cancer datasets available in the “Dataset selection” pane were added to the analysis. The |Log2FC| of 1 and the *p*-value cut-off of 0.01 were selected as the parameters. The option “Match TCGA normal and GTEx data” was also chosen. Therefore, TCGA cancer transcriptomics data were either matched with TCGA normal data or with data from the Genotype-Tissue Expression (GTEx) project, which is a public database containing tissue-specific gene expression and regulation data from 54 healthy tissue sites across more than 1000 individuals. The method for differential analysis is one-way ANOVA, using disease state (tumor or normal) as the variable for calculating differential expression. Only the statistically significant genes were used for further analyses.

### 2.2. Survival Analysis

The neurotransmitter receptor genes were also inputted into the XENA browser [37] (www.xenabrowser.net, accessed on 22 January 2021), where each TCGA tumor analyzed using GEPIA2 was accessed: (1) the TCGA pan-cancer (PANCAN) study was selected; (2) the “Genomic” data type was selected as the “first variable”; (3) the DE genes identified for each cancer type were inputted; and (4) the gene expression was selected. The normalized RNA-seq data were also downloaded as a spreadsheet file. Gene expression data accompanied by the survival data and clinicopathological annotation were downloaded from XENA and imported into Excel for further data curation. The selected data were then imported into R (version 4.0.2) and RStudio (version 1.3.1073) where: (1) an optimal cut-off score, defined as the gene expression level with the most significant split using a log-rank test, was selected to demonstrate the prognostic performance of each gene. The optimal cut-off was computed using the “maxstat.test” function (“maxstat” package [38]). (2) Patients were then stratified into low- and high-expression groups applying the optimal cut-off to the gene expression values. (3) The prognostic value of each gene was evaluated using Kaplan−Meier (KM) curves. The “survival” package in R/Bioconductor [39] was used to conduct the survival analysis. The KM curves were considered statistically significant when the log-rank *p* ≤ 0.05. (4) The effect on the overall survival probabilities of each NTR gene was assessed using the Cox proportional-hazard model. The assumption of the model was evaluated using the scaled Schoenfeld residual test through the “cox.zph” function (survival package [39] in R/Bioconductor).

### 2.3. Construction of the Cancer-Specific NTR Gene Panels and Prognostic Indexes

The NTR-associated LGG and GBM prognostic indexes were computed as $\underset{i}{\sum }Expression(Gene_{i})\cdot Beta_{i}$. In both cases, the prognostic indexes were used to stratify the patients into risk groups and their prognostic value was estimated by KM plots and applying a Cox proportional-hazard model. The assumption of the model was checked through the scaled Schoenfeld residual (using the “cox.zph” function available in the R/Bioconductor survival package [39]). These two prognostic indexes were further tested on two independent GEO (Gene Expression Omnibus) datasets: GSE107850 for LGG and GSE4412-GPL96 for GBM. These two datasets were both accessed using Phantasus (v.1.9.2, https://artyomovlab.wustl.edu/phantasus/, accessed on 25 January 2021), gene expression profiles were normalized, log2-transformed, collapsed, and exported into spreadsheet files for further analyses. The dataset was collected using the platform Illumina HumanHT-12 WG-DASL V4.0 R2 expression Beadchip, and the Affymetrix Human Genome U133A Array for the dataset GSE4412-GPL96.

### 2.4. Gene Ontology (GO) Enrichment Analysis

The GO enrichment analysis of the 10 NTR genes that exhibited an opposite association with clinical outcomes in LGG and GBM was performed in Orange (Version 3.26). Significance was determined by a hypergeometric test (*p* = 0.01).

### 2.5. Correlation Analysis

The function “corrplot” from the package “corrplot” was used to display a matrix of the Pearson correlation. Negative and positive correlations are displayed in red and blue, respectively. The intensity of the color and the size of the dots are proportional to the correlation coefficients.

### 2.6. Network Analysis

The NetworkAnalyst (https://www.networkanalyst.ca, accessed on 25 January 2021) was used to perform the network analysis of the 10 NTR genes with opposite associations with clinical outcomes between LGG and GBM. Specifically, tissue-specific (whole brain) protein–protein interaction (PPI) analysis was selected.

### 2.7. Gene Expression Analysis between Normal Tissue, LGG, and GBM

To conduct this analysis, the TCGA-TARGET-GTEx dataset was accessed using the XENA browser. The mean expression levels of the 10-NTR, 36 immune-related and 12 inflammasomes genes were compared across samples from normal, LGG, and GBM tissues. Box plots were generated to show the differences in gene expression between each group using the “ggplot2” package (v3.3.3). A pairwise, two-sided, unpaired two-sample Wilcoxon test was performed among the “normal”, “LGG”, and “GBM” groups.

### 2.8. Protein Expression Analysis between Normal Tissue and GBM

The proteins encoded by the 10-NTR genes were inputted into the University of Alabama at Birmingham cancer data analysis portal (UALCAN). The expression levels of these proteins were compared between the GBM and normal tissue. The proteomics datasets belong to the Clinical Proteomic Tumor Analysis Consortium (CPTAC). No LGG dataset is currently available in this database. Seven out of the ten inputted proteins were found. Box plots were then visualized where on the y axis the Z-values were shown. The Z-values were the standard deviations from the medians across the samples for each group. The spectral count ratio values from CPTAC were first log2-transformed, normalized within each sample profile, and then normalized across samples. Lastly, two-sided *t*-tests between the two groups were computed.

## 3. Results

### 3.1. Identification of Differentially Expressed NT Receptors Genes in Brain Cancer Tissue

**Figure 1:**
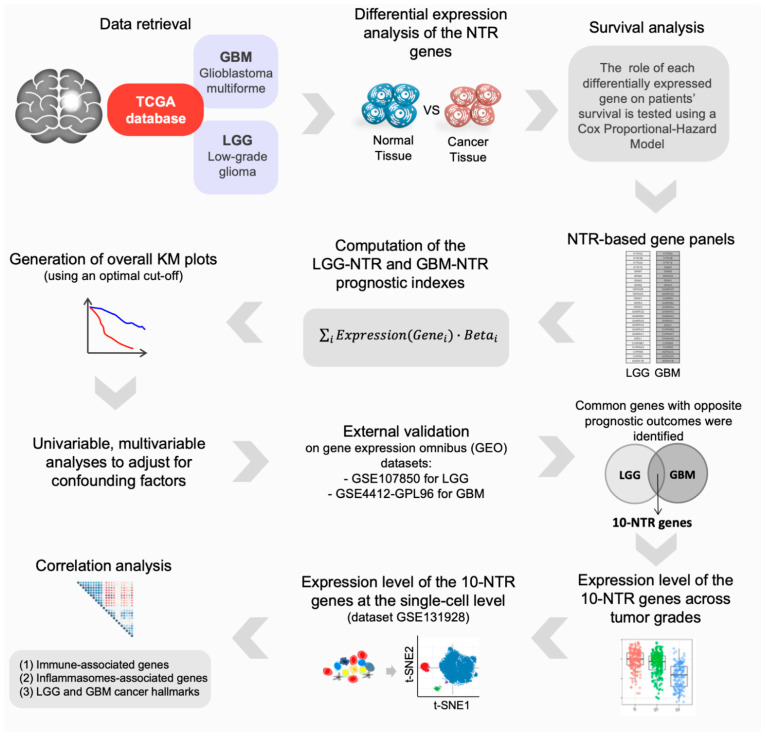
Schematic of the bioinformatic pipeline. We first identified the NTR genes that were differentially expressed in LGG and GBM tissue; then, we investigated their prognostic value. We constructed two gene panels and prognostic indexes (LGG-NTR and GBM-NTR) and we used them to stratify patients into risk groups. We performed univariable and multivariable analyses to adjust for confounding factors and validated both prognostic indexes on independent datasets. Next, we sought to find common genes between the two panels that had an opposite association with the patients’ survival and we analyzed their expression levels across progressive grades in two independent datasets as well as at the single-cell level. Finally, we evaluated the correlation between these genes (10-NTR) and genes associated with immune response, inflammasomes, and cancer hallmarks specific for LGG and GBM.

The workflow of the transcriptomic analysis is summarized in Figure 1. Genes encoding for classical neurotransmitter receptors (NTRs) were investigated. Specifically, using the HUGO Gene Nomenclature Committee (HGNC, www.genenames.org, accessed on 21 January 2021), 98 genes (Table S1) were identified. Next, we sought to identify which of the NT receptor genes were differentially expressed in each histological subtype of brain cancer that was available in the web-based tool GEPIA2 [40] with matched normal tissue. The details of the cancer types can be found in Table S2.

**Figure 2:**
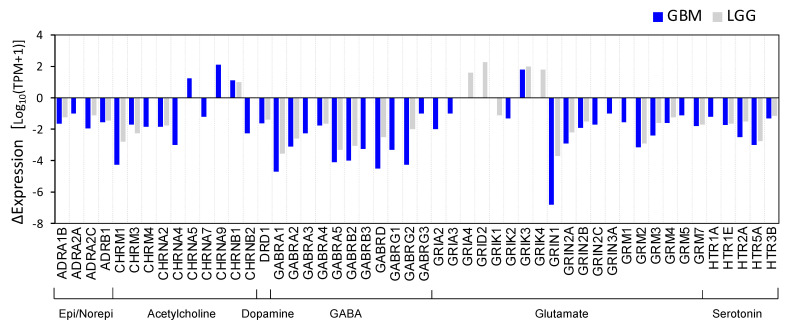
Expression of NTR genes in normal and cancer tissues. The bar plots represent the “ΔExpression = median expression (tumor) − median expression (normal)”. On the x-axis are the gene symbols. Both GBM *(N* = 163) and LGG *(N* = 518) were matched with normal brain samples *(N* = 207).

We found that 50 out of 98 genes were differentially expressed in brain cancer tissue. The results of the differential expression (DE) analysis are shown in Figure 2, where the differences in gene expression between cancer and normal tissues, measured from the outputs of the GEPIA2 “DEG analysis”, are plotted for each NTR gene. The NTR genes were under-expressed in tumor tissues relative to the matched normal tissues. Both GBM (*N* = 163) and LGG (*N* = 518) were matched with normal brain samples (*N* = 207).

### 3.2. Prognostic Impact of Differentially Expressed NTR Genes in LGG and GBM

**Figure 3:**
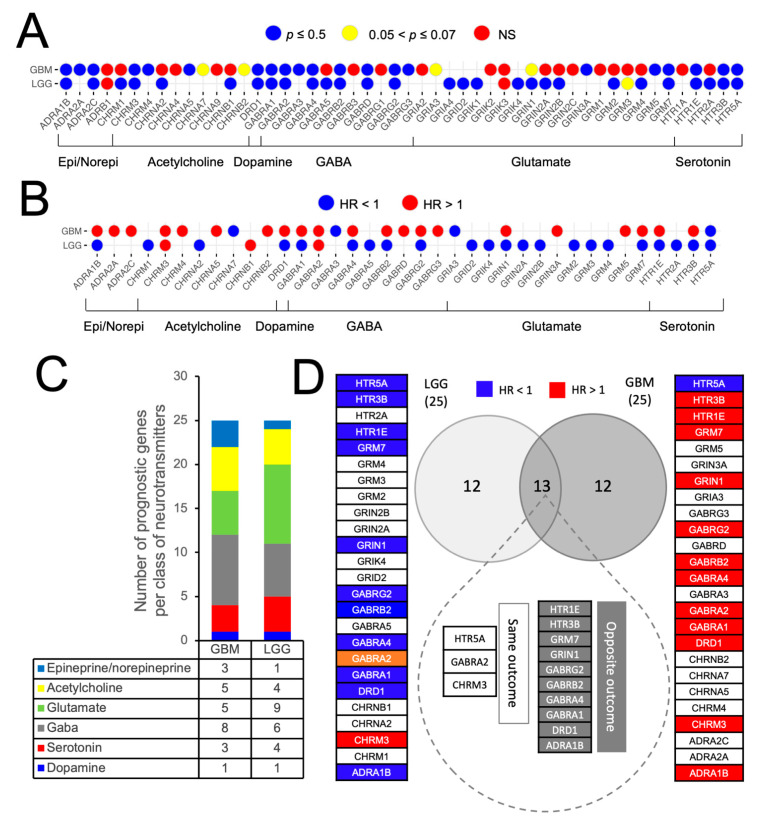
Impact of NTR genes on patient survival. (**A**) The color of the dots represents three different outcomes of the *p*-value of the log-rank test of each of the differentially expressed genes (DEGs) shown in Figure 2. On the x-axis, the gene symbols are shown, while the y-axis reports the symbols of the cancer types. The non-significant (NS) cases did not achieve statistical significance. (**B**) The color of the dots represents the value of the hazard ratio (HR) resulting from the application of the Cox model to each of the genes that reached statistical significance at the log-rank test in (**A**). The NTR genes associated with “negative prognosis” are in red, and the genes associated with “positive prognosis” are in blue. All the NTR genes that exhibited a *p* ≤ 0.05 at the scaled Schoenfeld residuals (Supplementary Table S2) test were excluded from the analysis. (**C**) Distribution of negative and positive DE prognostic NTR genes across the two cancer types, for each class of neurotransmitters. (**D**) Prognostic NTR genes shared by LGG and GBM and their impact on overall survival.

Next, we sought to understand whether the differentially expressed NTR genes had an impact on the patients’ survival. Kaplan–Maier (KM) plots were generated for each DEG, for each malignancy, as shown in Figures S1 and S2 for LGG and GBM, respectively. These results are summarized in Figure 3A. The NTR genes identified were segregated into three clusters based on the results of the log-rank test: (1) *p* ≤ 0.05; (2) 0.05 < *p* ≤ 0.07; (3) non-significant (NS). The prognostic value of each of these genes in Figure 3A was further tested using a Cox proportional-hazard model. The hazard ratio associated with each gene was calculated. The assumption of the proportional hazard model was tested using the scaled Schoenfeld residuals test. The impact of NTR gene expression across different glioma types on the overall survival is shown in Figure 3B, where the color of the dots represents positive (blue, HR < 1) or negative (red, HR > 1) association with the overall patients’ survival. Specifically, for GBM, the following genes had an HR < 1: *CHRNA7*, *GABRA3*, *GRIA3*, and *HTR5A*; whereas the following genes had an HR > 1: *ADRA1B*, *ADRA2A*, *ADRA2C*, *CHRM3*, *CHRM4*, *CHRNA5*, *CHRNB2*, *DRD1*, *GABRA1*, *GABRA2*, *GABRA4*, *GABRB2*, *GABRD*, *GABRG3*, *GRIN1*, *GRIN3A*, *GRM5*, *GRM7*, *HTR1E*, and *HTR3B*. For LGG, the following genes had an HR < 1: *ADRA1B*, *CHRM1*, *CHRNA2*, *DRD1*, *GABRA1*, *GABRA4*, *GABRA5*, *GABRB2*, *GABRG2*, *GRID2*, *GRIK4*, *GRIN1*, *GRIN2A*, *GRIN2B*, *GRM2*, *GRM3*, *GRM4*, *GRM7*, *HTR1E*, *HTR2A*, *HTR3B*, and *HTR5A*; whereas the following genes had an HR > 1: *CHRM3*, *CHRNB1*, and *GABRA2*. Figure 3C shows the number of the differentially expressed genes that also had a prognostic value for each class of neurotransmitters.

### 3.3. Identification and External Validation of NTR-Associated Gene Signatures

**Figure 4:**
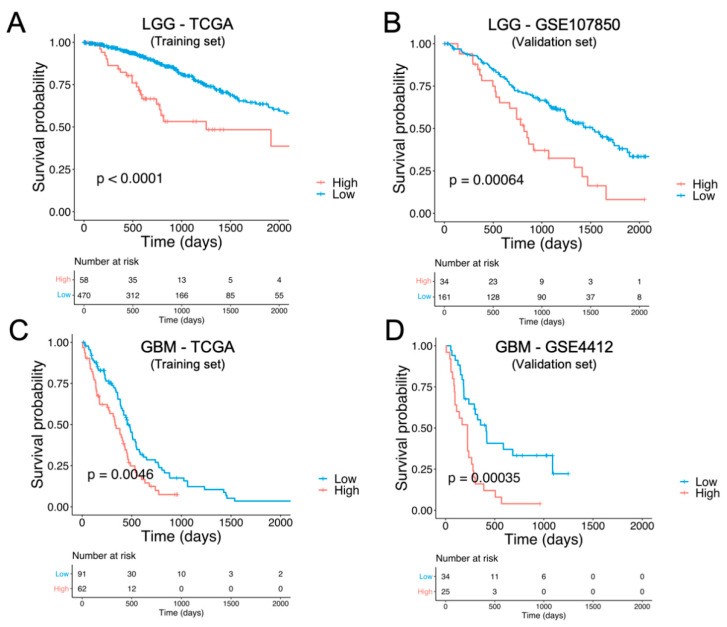
Prognostic value of the NTR gene signatures and their validation. (**A**) The overall survival (OS) curve of the gene signature generated using the LGG-NTR prognostic genes shown in Figure 3B. Blue and red KM curves represent the predicted low-risk and high-risk groups, respectively. The log-rank *p*-value and the number of patients at risk are shown for each risk group. (**B**) OS curve of the gene signature identified using the TCGA-LGG dataset in a GEO-independent dataset (GSE107859). (**C**) OS curve of the GBM-NTR gene signature generated by using the TCGA-GBM prognostic genes shown in Figure 3B. (**D**) OS curve of the gene signature identified using the TCGA-GBM dataset in a GEO-independent dataset (GSE4412-GPL96).

We next identified and validated NTR-associated gene signatures using independent datasets. First, we calculated the NTR-LGG prognostic index by multiplying the expression level by the hazard ratio of each gene and summing these values for all the prognostic NTR genes identified for LGG in Figure 3B. The same was completed in the case of GBM. These two gene signatures were then tested on two GEO (Gene Expression Omnibus) datasets: GSE107850 for LGG and GSE4412-GPL96 for GBM. In both cases, the NTR-associated gene signatures stratified the patients into low- and high-risk groups and the former had a statistically significant longer survival, as shown in Figure 4. Specifically, high values of the NTR-LGG index were associated with positive prognosis, whereas high values of the NTR-GBM index were associated with a negative prognosis, as a result of the HR of the genes included in each signature (Figure 3D). In order to adjust for confounding factors, a multivariable Cox regression analysis was performed (Figure S3). Moreover, for both LGG and GBM, most of the prognostic genes exhibited a strong positive correlation (Figure S4). Only one gene (CHRNB1) exhibited a moderate negative correlation, with almost all the other prognostic genes in LGG.

### 3.4. Identification of NTR Genes with Opposite Prognostic Outcomes in LGG and GBM

**Figure 5:**
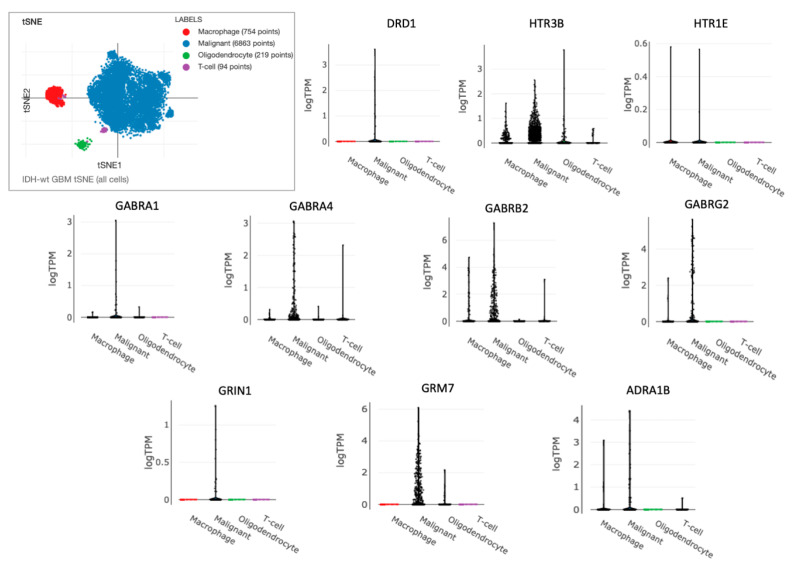
Single-cell gene expression levels of the 10-NTR genes in GBM. The single-cell RNA-seq GBM dataset (GEO accession number GSE131928) was accessed using the Single Cell Portal (https://singlecell.broadinstitute.org/single_cell, accessed on 20 April 2021). In the upper corner, the t-distributed stochastic neighbor embedding (tSNE) plot of all single cells from 28 glioblastomas is shown. Four clusters were identified which were associated with the high expression of markers for malignant cells and three non-malignant cell types. The violin plots show the single-cell gene expression levels for each cell cluster.

Figure 3D shows that 13 DE prognostic genes were shared between LGG and GBM (*HTR5A*, *HTR3B*, *HTR1E*, *GRM7*, *GRIN1*, *GABRG2*, *GABRB2*, *GABRA4*, *GABRA2*, *GABRA1*, *DRD1*, *CHRM3*, and *ADRA1B*). Three of them (*HTR5A*, *GABRA2*, and *CHRM3*) were associated with the same clinical outcomes in both cancers, whereas the remaining 10 genes (*DRD1*, *HTR1E*, *HTR3B*, *GABRA1*, *GABRA4*, *GABRB2*, *GABRG2*, *GRIN1*, *GRM7*, and *ADRA1B*) exhibited an opposite association with clinical outcomes. Specifically, these genes predicted positive prognosis in LGG and negative prognosis in GBM. We then explored how the expression levels of this 10-gene panel, which we defined as “10-NTR”, varied between normal tissues and across progressive WHO grades. For the former analysis, we used GEPIA2 to access the TCGA and GTEx datasets, whereas for the latter analysis we accessed both the TCGA and the CGGA (Chinese Glioma Genome Atlas) databases using GlioVis [41] and sorted the data as a function of the WHO grade. For all the 10-NTR genes, the expression levels tended to decrease progressively as the grade increased from grade II (low-grade glioma, *N* = 515 for TCGA and *N* = 625 for CGGA) to IV (glioblastoma multiforme, *N* = 152 for TCGA and *N* = 338 for CGGA), as shown in Figure 5. The gene HTR3B was not available in the CGGA dataset.

The top 10 enrichment scores in the gene ontology (GO) enrichment analysis of the 10-NTR genes are shown in Figure S6A, whereas the brain-specific protein–protein interaction gene network is shown in Figure S6B, and the KEGG analysis indicates the most enriched pathways (*p* ≤ 0.01) involving the genes in the network in Table S3.

Furthermore, we performed gene expression analysis of the 10-NTR genes on a single-cell RNA-seq GBM dataset (GEO accession number GSE131928) from Neftel et al. [42] that was accessed using the Single Cell Portal. Across the main clusters of cells identified by Neftel et al. [42], we found that the cells with the highest expression of these 10 genes were the malignant cells, as shown in Figure 5. The genes with the highest expression were *HTR3B*, *GABRA4*, *GABRB2*, *GABRG2*, *GRM7*, whereas *DRD1*, *HTR1E*, and *GRIN1* had very low expression levels in this dataset.

### 3.5. Association between the 10-NTR Genes and Immune- and Inflammasome-Associated Markers

**Figure 6:**
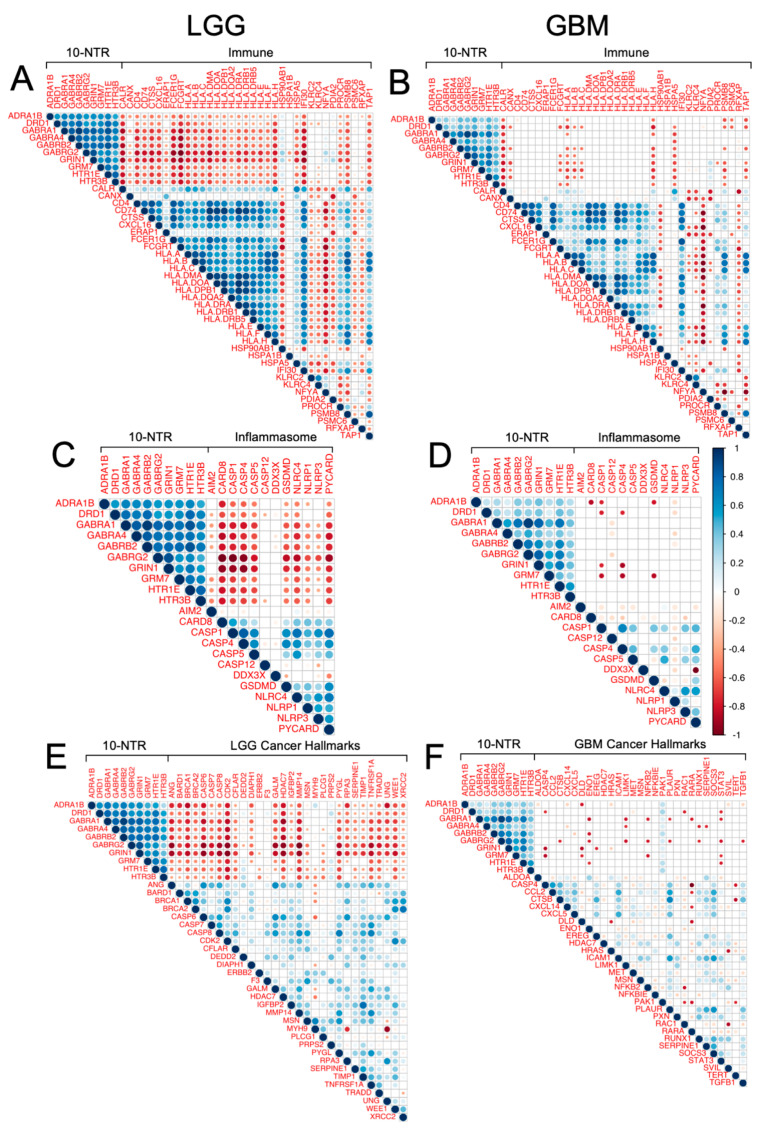
Correlation analysis between the 10-NTR genes and genes associated with immune response, inflammasomes, and cancer hallmarks. (**A**) Correlation between the 10-NTR genes and 36 immune-related genes (IRGs) in LGG. (**B**) Correlation between the 10-NTR genes and 36 IRGs in GBM. (**C**) Correlation between the 10-NTR genes and the inflammasomes genes. (**D**) Correlation between the 10-NTR genes and the 12 inflammasomes genes in GBM. (**E**) Correlation between the 10-NTR genes and LGG-specific hallmarks. (**F**) Correlation between the 10-NTR genes and GBM-specific hallmarks. Color intensity and size of the dots are proportional to the Pearson correlation coefficients. Blank spaces correspond to non-significant coefficients.

Furthermore, 36 immune-related genes (IRGs) with prognostic values in LGG have been recently identified by Zhang et al. [43]: *CALR*, *CANX*, *CD4*, *CD74*, *CTSS*, *FCER1G*, *FCGRT*, *HLA-A*, *HLA-B*, *HLA-C*, *HLA-DMA*, *HLA-DOA*, *HLA-DPB1*, *HLA-DQA2*, *HLA-DRA*, *HLA-DRB1*, *HLA-DRB5*, *HLA-E*, *HLA-F*, *HLA-H*, *HSPA1B*, *HSPA5*, *HSP90AB1*, *KLRC2*, *NFYA*, *PSMB8*, *PSMC6*, *RFXAP*, *TAP1*, *TAPBC*, *KLRC4*, *IFI30*, *PROCR*, *ERAP1*, *PDIA2*, and *CXCL16.* We tested the correlation between the 10-NTR genes and the IRGs, both in LGG and GBM. In Figure 6A,B, the correlation between the 10 NTR genes and the 36 IRGs is shown for both cancer types. A strong negative correlation was observed in the case of LGG between the NTR genes and the IRGs associated with adverse prognostic outcomes in the work of Zhang et al. [43]. The correlation pattern became substantially weaker in the case of GBM, where most of the correlations were no longer statistically significant.

Moreover, we tested the correlation between the 10-NTR genes and 12 inflammasomes genes [44]: *AIM2*, *CARD8*, *CASP1*, *CASP4*, *CASP5*, *CASP12*, *DDX3X*, *GSDMD*, *NLRC4*, *NLRP1*, *NLRP3*, and *PYCARD*. A strong negative correlation was found between most of the genes composing these two panels in the case of LGG (Figure 6C). In the case of GBM, as for the IRGs, most of the correlations were no longer statistically significant (Figure 6D).

### 3.6. Association between 10-NTR Genes and Cancer-Specific Hallmark Genes

We sought to test whether these 10 genes had any interactions with established cancer-specific hallmarks for both LGG and GBM. The cancer-specific hallmark genes were sourced from recent work by Nagy et al. [45]. Specifically, the first 30 genes with log-rank *p* ≤ 0.01 and HR > 1 were selected for both cancer types. In the case of LGG, the selected genes were: *WEE1*, *IGFBP2*, *UNG*, *CASP8*, *TIMP1*, *RPA3*, *CASP6*, *CFLAR*, *BRCA1*, *PYGL*, *MSN*, *CASP7*, *PRPS2*, *TNFRSF1A*, *PLCG1*, *CDK2*, *DIAPH1*, *BRCA2*, *ANG*, *F3*, *XRCC2*, *GALM*, *MMP14*, *DEDD2*, *ERBB2*, *HDAC7*, *TRADD*, *MYH9*, *SERPINE1*, *and BARD1*; whereas for GBM, the selected genes were: *PAK1*, *PXN*, *ENO1*, *CTSL*, *CXCL14*, *CCL2*, *HDAC7*, *SVIL*, *LIMK1*, *SOCS3*, *HRAS*, *ICAM1*, *PLAUR*, *RAC1*, *EREG*, *CXCL5*, *CTSB*, *RUNX1*, *SERPINE1*, *CASP4*, *NFKBIE*, *TGFB1*, *MET*, *MSN*, *NFKB2*, *STAT3*, *RARA*, *DLD*, *TERT*, and *ALDOA*. In Figure 6E,F, the correlation between the 10-NTR genes and the cancer-specific hallmarks genes is shown for LGG and GBM, respectively. Most of the 10-NTR genes were strongly negatively correlated with the top 30 LGG-hallmarks, whereas most of the correlations were not statistically significant in the case of GBM.

**Figure 7:**
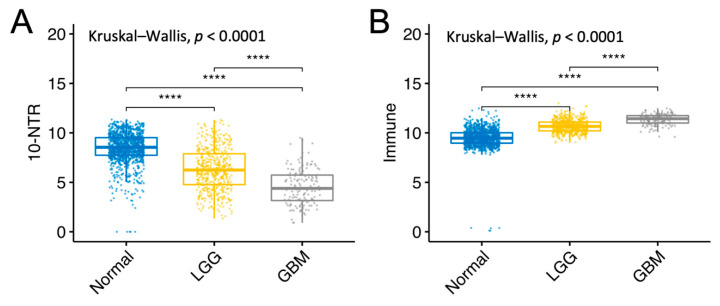

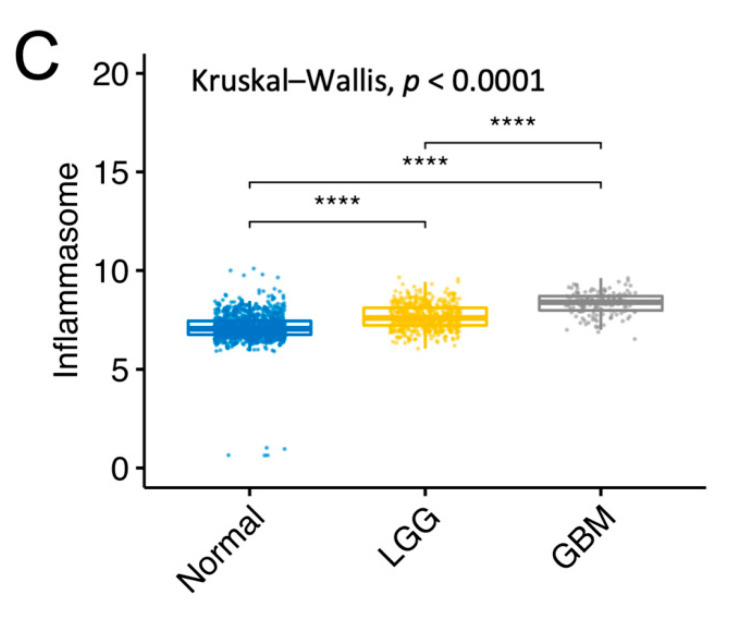
Gene expression levels of the 10-NTR, immune, and inflammasome gene panels in normal, LGG, and GBM tissue. (**A**) The average expression of the 10-NTR genes is plotted against different tissue types: normal, LGG and GBM. (**B**) Average expression of the 36 immune-related genes (IRGs) in the three tissue types. (**C**) Average expression of the 12 inflammasome genes in the three tissue types. Wilcoxon test **** *p* ≤ 0.0001. Expression levels are measured in Log2 (normalized count + 1).

Figure 7 shows the change in gene expression of the 10-NTR-, immune-, and inflammasome-associated signatures moving from normal tissue to LGG and GBM. Specifically, a statistically significant difference in the expression level of the 10-NTR genes was found between normal tissue and LGG, LGG and GBM, and GBM and normal tissue, as shown in Figure 7A. Similar results were found at the protein level when comparing the expression levels in normal tissue and GBM. Specifically, statistically significant differences were found between the two groups, as shown in Figure S8. Moreover, a statistically significant difference in the expression level of the immune-related genes was found between normal tissue and LGG, LGG and GBM, and GBM and normal tissue, as shown in Figure 7B. Furthermore, a statistically significant difference in the expression level of the inflammasome-related genes was found between normal tissue and LGG, LGG and normal tissue, and GBM and normal tissue, as shown in Figure 7C. Finally, for both LGG and GBM, a strong positive correlation was found between the immune and inflammasome panels, as shown in Figure S7.

## 4. Discussion

This study systematically investigated the impact of neurotransmitter receptors on brain cancer progression by utilizing the largest publicly available cancer database, comprised of 618 individuals, from The Cancer Genome Atlas (TCGA) dataset. Our results provide a landscape of the dysregulated genes encoding for NT receptors and their impact on brain cancer patients’ overall survival in low-grade glioma and glioblastoma multiforme.

A differential expression of a subset of NTR genes was found when comparing cancer and normal brain tissues. Specifically, we found that the majority of the differentially expressed NTR genes were under-expressed in both LGG and GBM. This could be possibly explained by the downregulation of neurotransmitter receptors via receptor-ligand-induced desensitization [46], resulting from the elevated levels of the neurotransmitters present in the tumor microenvironment compared to normal tissue. This was recently demonstrated by Nguyen et al. [47] for the peptide-based neurotransmitter N-acetyl-aspartyl-glutamate (NAAG). Further studies are required to determine cause and effect in relation to cancer cells and the dysregulated matrisomal components.

Of the NTR genes which had prognostic values for LGG and GBM, most of them belonged to the following classes: acetylcholine, glutamate, and GABA. Two 25-gene NTR-associated signatures allowed us to stratify brain cancer patients into low and high-risk groups within LGG and GBM, independently. For both cancer types, the prognostic genes strongly correlated with each other, suggesting that biological associations among neurotransmitters of different classes exist. A subset of 13 prognostic genes was found to be shared by LGG and GBM, 10 of which (*DRD1*, *HTR1E*, *HTR3B*, *GABRA1*, *GABRA4*, *GABRB2*, *GABRG2*, *GRIN1*, *GRM7*, and *ADRA1B*) were associated with favorable prognostics for LGG, but were unfavorable for GBM. This suggests that the direct or indirect involvement of these 10 NTR genes might exist in the progression from LGG to GBM. The decreasing expression levels of these genes across successive histology grades are presumably associated with a decreased density of nerve fiber infiltration in the tumor microenvironment moving from low to higher histology grades. Previous studies have shown that white matter fiber infiltration tends to be destroyed in higher-grade tumors [48,49] compared to low-grade tumors. Moreover, using diffuse tensor imaging (DTI), Davanian et al. [50] found that fiber tracts in the proximity of low-grade gliomas are intact and well-organized, while peritumoral fiber tracts in high-grade gliomas are damaged and disorganized. It is worth noting that 4 out of the 10 NTR genes encoded for GABA receptors, suggesting that this class of NTs merits further investigations in GBM. GABA receptors are expressed in low-grade glioma cells but not in higher-grade gliomas [51] and glioma cell lines without GABA receptor expression exhibited unlimited proliferation in culture [52], suggesting a possible regulatory role. The role of other classes of NTRs on glioma cells’ proliferation requires further investigation. To better understand which cell types contribute to the observed gene expression and to account for the inherent heterogeneity of a tissue biopsy, we looked at a single-cell RNA-seq GBM dataset. Specifically, we found a high expression of “10-NTR” genes in the cancer cells and macrophages (Figure 5). Accumulating evidence regarding the interactions between neurotransmitters and inflammation is emerging, with important consequences for the regulation of the immune response. For instance, it has been shown that, in rodents, GABA can reduce the release of inflammatory cytokines through the inhibition of the NF-kB and p38 MAPK signaling pathways [53]. Serotonin and its receptors have been shown to have an important role in the activation of immune responses and inflammation [54]. Glutamate has been shown to modulate T cell-mediated immune responses [55]. Dopamine has been reported to inhibit inflammasomes [56]. Epinephrine, norepinephrine, and acetylcholine have been demonstrated to play anti-inflammatory roles [57,58]. Therefore, our results on the negative correlations between the 10-NTR genes and those associated with inflammasomes and immune response in LGG, which are in line with the aforementioned literature, appear to suggest a protective role of these NTR genes in lower-grade gliomas. Our further findings of these genes becoming progressively downregulated in higher grades could presumably highlight the importance of these genes in preventing the malignant progression of the tumor. This phenomenon is likely to result from the dysregulation of a complex equilibrium among all the components of the tumor microenvironment and the bidirectional interactions among them. More studies are needed to investigate causal relationships that orchestrate malignant progression in gliomas. It is noteworthy that, for GBM, despite the lack of correlation found between the 10-NTR genes and the genes associated with immune and inflammation, the latter markers appeared to be strongly correlated with each other, similarly to the case of LGG (Figure S7B).

Finally, we investigated the correlation between the 10-NTR genes and the brain cancer-specific hallmarks recently published by Nagy et al. [45]. Most of the 10-NTR genes were strongly negatively correlated with the top 30 LGG-hallmark genes, highlighting the role of the 10-NTR genes as positive prognostic factors. The correlation pattern was substantially different in the case of 30 GBM-hallmark genes, where most of their correlations were not statistically significant. This could presumably be due to a higher upregulation of the hallmark genes in GBM, as a consequence of its higher degree of transformation as compared with LGG. Hence, the hallmark genes would become dominant in GBM and the correlation between their expression levels and those of the 10-NTR genes would become negligible. Notably, the gene *PAK1* was positively correlated with all the 10-NTR genes. This gene was previously identified as an unfavorable prognostic marker in glioma and encodes for a protein kinase involved in cell adhesion, cell migration, proliferation, mitosis, cytoskeleton dynamics, apoptosis, and vesicle-mediated transport processes.

This study is limited to the investigation of classical NT receptors, and did not include neuropeptides, which are released by nerves in both the central and peripheral nervous system and play an important role in mediating the communication between the nervous system and the tumor microenvironment [59]. Specifically, neuropeptide Y has been shown to increase angiogenesis in xenograft models of neural-crest-derived tumors [60], whereas neurotensin, bombesin, and endothelin-1 are associated with the regulation of the survival and migratory functions of androgen-independent prostate cancer [61,62,63]. Therefore, future studies should focus on the role of neuropeptides in the progression of gliomas.

## 5. Conclusions

Taken together, this study confirms that neurotransmitters and their receptors have an important role in tumor progression and survival—as observed in recent studies on the role of acetylcholine [6] and dopamine [64] receptors—and extends analysis to all the classical neurotransmitter receptors. Additionally, this study identifies a subset of NTR genes as an emerging hallmark of brain gliomas and highlights the existence of a strong interplay between inflammation, neurotransmitters, and immune response at the basis of the malignant progression of human gliomas. These results might have implications for the development of novel therapeutics and preventive strategies that target regulatory networks associated with the link between the autonomic nervous system, cancer cells, and the tumor microenvironment. Future longitudinal studies should explore avenues for developing targeted therapies, for instance, classical drugs, such as serotonin receptor antagonists, dopamine agonists, and acetylcholine receptor antagonists, which might have clinical implications in cancer treatment [23,65]. Moreover, further studies are needed to investigate the link between neurotransmitters, gut microbiota, and other environmental factors such as nutrition, which have already been demonstrated in the field of brain disorders and illnesses [66,67,68], and interesting results are likely to emerge.

## Data Availability

TCGA (The Cancer Genome Atlas) data were accessed using the XENA browser (www.xenabrowser.net, accessed on 22 January 2021). GEO (Gene Expression Omnibus) datasets: GSE107850 for LGG (low-grade glioma) and GSE4412-GPL96 for Glioblastoma Multiforme (GBM) were both accessed using Phantasus (v.1.9.2, https://artyomovlab.wustl.edu/phantasus/, accessed on 22 January 2021). The CGGA (Chinese Glioma Genome Atlas) datasets were accessed using GlioVis (http://gliovis.bioinfo.cnio.es/, accessed on 25 January 2021). The Glioblastoma single-cell RNA-seq from Müller et al. [69] dataset was accessed using UCSC Cell Browser (Version: v1.0.0). The differential gene expression analysis was conducted in GEPIA2 (http://gepia2.cancer-pku.cn/, accessed on 21 January 2021). The optimal cut-off was computed in R using the “maxstat.test” function (“maxstat” package). The survival analysis was conducted using the “survival” package in R/Bioconductor. Dot plots were generated in R using “ggballoonplot” and the packages “ggplot2” and “ggpubr”.

## Supplementary Materials

The following supporting information can be downloaded at: https://www.mdpi.com/article/10.3390/cancers14102544/s1, Table S1: Details of the neurotransmitter receptor genes included in the analysis; Table S2: Outcomes of the univariate analysis using the Cox proportional-hazard model; Table S3: KEGG enrichment analysis; Figure S1. Overall survival probabilities as a function of the levels of the differentially expressed genes in LGG; Figure S2. Overall survival probabilities as a function of the levels of the differentially expressed genes in GBM; Figure S3. External validation of the prognostic value of the gene signatures identified in the TCGA brain cancers; Figure S4. Correlation among differentially expressed prognostic NTR genes in brain cancers; Figure S5. Gene expression levels of the 10-NTR genes as a function of the cancer grade; Figure S6. Gene ontology enrichment and network analysis of the 10-NTR genes; Figure S7. Correlation between immune and inflammasome gene panels in brain cancers. Figure S8. Protein expression levels of the NTR genes in GBM and normal tissue.
