# Supplementary material for: Prognostic Neurotransmitter Receptors Genes Are Associated with Immune Response, Inflammation and Cancer Hallmarks in Brain Tumors
Source: Cancers (Basel). 2022 May 21;14(10):2544. doi: 10.3390/cancers14102544 (PMC9139273; doi:10.3390/cancers14102544)
Supplement: Supplementary file 1 [file cancers-14-02544-s001.zip › cancers-1741759-supplementary.pdf]

Supplementary Information

Table S1. Details of the neurotransmitter receptor genes included in the analysis.

Class	HGNC ID (gene)	Approved symbol	Tumours in which the gene is DE	Approved name	Chromosome
Dopamine	HGNC:3020	DRD1	GBM, LGG	dopamine receptor D1	5q35.2
	HGNC:3023	DRD2	None	dopamine receptor D2	11q23.2
	HGNC:3024	DRD3	None	dopamine receptor D3	3q13.31
	HGNC:3025	DRD4	None	dopamine receptor D4	11p15.5
	HGNC:3026	DRD5	None	dopamine receptor D5	4p16.1
Serotonin	HGNC:5286	HTR1A	GBM	5-hydroxytryptamine receptor 1A	5q12.3
	HGNC:5287	HTR1B	None	5-hydroxytryptamine receptor 1B	6q14.1
	HGNC:5289	HTR1D	None	5-hydroxytryptamine receptor 1D	1p36.12
	HGNC:5291	HTR1E	GBM, LGG	5-hydroxytryptamine receptor 1E	6q14.3
	HGNC:5292	HTR1F	None	5-hydroxytryptamine receptor 1F	3p12
	HGNC:5293	HTR2A	GBM, LGG	5-hydroxytryptamine receptor 2A	13q14.2
	HGNC:5294	HTR2B	None	5-hydroxytryptamine receptor 2B	2q37.1
	HGNC:5295	HTR2C	None	5-hydroxytryptamine receptor 2C	Xq23
	HGNC:5299	HTR4	None	5-hydroxytryptamine receptor 4	5q32
	HGNC:5300	HTR5A	GBM, LGG	5-hydroxytryptamine receptor 5A	7q36.2
	HGNC:16291	HTR5BP	None	5-hydroxytryptamine receptor 5B, pseudogene	2q14.1
	HGNC:5301	HTR6	None	5-hydroxytryptamine receptor 6	1p36.13
	HGNC:5302	HTR7	None	5-hydroxytryptamine receptor 7	10q23.31
	HGNC:5297	HTR3A	None	5-hydroxytryptamine receptor 3A	11q23.2
	HGNC:5298	HTR3B	GBM, LGG	5-hydroxytryptamine receptor 3B	11q23.2
	HGNC:24003	HTR3C	None	5-hydroxytryptamine receptor 3C	3q27.1
	HGNC:24004	HTR3D	None	5-hydroxytryptamine receptor 3D	3q27.1
	HGNC:24005	HTR3E	None	5-hydroxytryptamine receptor 3E	3q27.1
GABA	HGNC:4075	GABRA1	GBM, LGG	gamma-aminobutyric acid type A receptor subunit alpha1	5q34
	HGNC:4076	GABRA2	GBM, LGG	gamma-aminobutyric acid type A receptor subunit alpha2	4p12
	HGNC:4077	GABRA3	GBM	gamma-aminobutyric acid type A receptor subunit alpha3	Xq28
	HGNC:4078	GABRA4	GBM, LGG	gamma-aminobutyric acid type A receptor subunit alpha4	4p12
	HGNC:4079	GABRA5	GBM, LGG	gamma-aminobutyric acid type A receptor subunit alpha5	15q12
	HGNC:4080	GABRA6	None	gamma-aminobutyric acid type A receptor subunit alpha6	5q34
	HGNC:4081	GABRB1	None	gamma-aminobutyric acid type A receptor subunit beta1	4p12
	HGNC:4082	GABRB2	GBM, LGG	gamma-aminobutyric acid type A receptor subunit beta2	5q34
	HGNC:4083	GABRB3	GBM	gamma-aminobutyric acid type A receptor subunit beta3	15q12
	HGNC:4084	GABRD	GBM, LGG	gamma-aminobutyric acid type A receptor subunit delta	1p36.33
	HGNC:4085	GABRE	None	gamma-aminobutyric acid type A receptor subunit epsilon	Xq28
	HGNC:4086	GABRG1	GBM	gamma-aminobutyric acid type A receptor subunit gamma1	4p12
	HGNC:4087	GABRG2	GBM, LGG,	gamma-aminobutyric acid type A receptor subunit gamma2	5q34
	HGNC:4088	GABRG3	GBM	gamma-aminobutyric acid type A receptor subunit gamma3	15q12
	HGNC:4089	GABRP	None	gamma-aminobutyric acid type A receptor subunit pi	5q35.1
	HGNC:14454	GABRQ	None	gamma-aminobutyric acid type A receptor subunit theta	Xq28
	HGNC:4090	GABRR1	None	gamma-aminobutyric acid type A receptor subunit rho1	6q15
	HGNC:4091	GABRR2	None	gamma-aminobutyric acid type A receptor subunit rho2	6q15
	HGNC:17969	GABRR3	None	gamma-aminobutyric acid type A receptor subunit rho3	3q11.2
Glutamate	HGNC:4571	GRIA1	None	glutamate ionotropic receptor AMPA type subunit 1	5q33.2
	HGNC:4572	GRIA2	GBM	glutamate ionotropic receptor AMPA type subunit 2	4q32.1
	HGNC:4573	GRIA3	GBM	glutamate ionotropic receptor AMPA type subunit 3	Xq25
	HGNC:4574	GRIA4	LGG	glutamate ionotropic receptor AMPA type subunit 4	11q22.3
	HGNC:4575	GRID1	None	glutamate ionotropic receptor delta type subunit 1	10q23.1-q23.2
	HGNC:4576	GRID2	LGG	glutamate ionotropic receptor delta type subunit 2	4q22.1-q22.2
	HGNC:4579	GRIK1	LGG	glutamate ionotropic receptor kainate type subunit 1	21q21.3
	HGNC:4580	GRIK2	GBM	glutamate ionotropic receptor kainate type subunit 2	6q16.3
	HGNC:4581	GRIK3	GBM, LGG	glutamate ionotropic receptor kainate type subunit 3	1p34.3
	HGNC:4582	GRIK4	LGG	glutamate ionotropic receptor kainate type subunit 4	11q23.3
	HGNC:4583	GRIK5	None	glutamate ionotropic receptor kainate type subunit 5	19q13.2
	HGNC:4584	GRIN1	GBM, LGG	glutamate ionotropic receptor NMDA type subunit 1	9q34.3
	HGNC:4585	GRIN2A	GMB, LGG	glutamate ionotropic receptor NMDA type subunit 2A	16p13.2
	HGNC:4586	GRIN2B	GMB, LGG	glutamate ionotropic receptor NMDA type subunit 2B	12p13.1
	HGNC:4587	GRIN2C	GBM	glutamate ionotropic receptor NMDA type subunit 2C	17q25.1
	HGNC:4588	GRIN2D	None	glutamate ionotropic receptor NMDA type subunit 2D	19q13.33
	HGNC:16767	GRIN3A	GBM	glutamate ionotropic receptor NMDA type subunit 3A	9q31.1
	HGNC:16768	GRIN3B	None	glutamate ionotropic receptor NMDA type subunit 3B	19p13.3
	HGNC:4593	GRM1	GBM	glutamate metabotropic receptor 1	6q24.3
	HGNC:4594	GRM2	GBM, LGG	glutamate metabotropic receptor 2	3p21.2
	HGNC:4595	GRM3	GBM, LGG	glutamate metabotropic receptor 3	7q21.11-q21.12
	HGNC:4596	GRM4	GBM, LGG	glutamate metabotropic receptor 4	6p21.31
	HGNC:4597	GRM5	GBM	glutamate metabotropic receptor 5	11q14.2-q14.3
	HGNC:4598	GRM6	None	glutamate metabotropic receptor 6	5q35.3
	HGNC:4599	GRM7	GBM, LGG	glutamate metabotropic receptor 7	3p26.1
	HGNC:4600	GRM8	None	glutamate metabotropic receptor 8	7q31.33
Acetylcholine	HGNC:1950	CHRM1	GBM, LGG	cholinergic receptor muscarinic 1	11q12.3
	HGNC:1951	CHRM2	None	cholinergic receptor muscarinic 2	7q33
	HGNC:1952	CHRM3	GBM, LGG	cholinergic receptor muscarinic 3	1q43
	HGNC:1953	CHRM4	GBM	cholinergic receptor muscarinic 4	11p11.2
	HGNC:1954	CHRM5	None	cholinergic receptor muscarinic 5	15q14
	HGNC:1955	CHRNA1	None	cholinergic receptor nicotinic alpha 1 subunit	2q31.1
	HGNC:1956	CHRNA2	GBM, LGG	cholinergic receptor nicotinic alpha 2 subunit	8p21.2
	HGNC:1957	CHRNA3	None	cholinergic receptor nicotinic alpha 3 subunit	15q25.1
	HGNC:1958	CHRNA4	GBM	cholinergic receptor nicotinic alpha 4 subunit	20q13.33
	HGNC:1959	CHRNA5	GBM	cholinergic receptor nicotinic alpha 5 subunit	15q25.1
	HGNC:15963	CHRNA6	None	cholinergic receptor nicotinic alpha 6 subunit	8p11.21
	HGNC:1960	CHRNA7	GBM	cholinergic receptor nicotinic alpha 7 subunit	15q13.3
	HGNC:14079	CHRNA9	GMB	cholinergic receptor nicotinic alpha 9 subunit	4p14
	HGNC:13800	CHRNA10	None	cholinergic receptor nicotinic alpha 10 subunit	11p15.4
	HGNC:1961	CHRNB1	GBM, LGG	cholinergic receptor nicotinic beta 1 subunit	17p13.1
	HGNC:1962	CHRNB2	GBM	cholinergic receptor nicotinic beta 2 subunit	1q21.3
	HGNC:1963	CHRNB3	None	cholinergic receptor nicotinic beta 3 subunit	8p11.21
	HGNC:1964	CHRNB4	None	cholinergic receptor nicotinic beta 4 subunit	15q25.1
	HGNC:1965	CHRND	None	cholinergic receptor nicotinic delta subunit	2q37.1
	HGNC:1966	CHRNE	None	cholinergic receptor nicotinic epsilon subunit	17p13.2
	HGNC:1967	CHRNG	None	cholinergic receptor nicotinic gamma subunit	2q37.1
Epi/Norepi	HGNC:277	ADRA1A	None	adrenoceptor alpha 1A	8p21.2
	HGNC:278	ADRA1B	GBM, LGG	adrenoceptor alpha 1B	5q33.3
	HGNC:280	ADRA1D	None	adrenoceptor alpha 1D	20p13
	HGNC:281	ADRA2A	GBM	adrenoceptor alpha 2A	10q25.2
	HGNC:282	ADRA2B	None	adrenoceptor alpha 2B	2q11.2
	HGNC:283	ADRA2C	GBM, LGG	adrenoceptor alpha 2C	4p16.3
	HGNC:285	ADRB1	GBM, LGG	adrenoceptor beta 1	10q25.3
	HGNC:286	ADRB2	None	adrenoceptor beta 2	5q32
	HGNC:288	ADRB3	None	adrenoceptor beta 3	8p11.23

Table S2. Outcomes of the univariate analysis using the Cox proportional-hazard model. The prognostic value of each of the genes in Fig. 3a was tested using a univariate analysis. The assumption of the proportional hazard model was tested using the scaled Schoenfeld residuals test and the P-values are shown in the tables.

TCGA cancer	Gene symbol	HR (95% CI)	p value
GBM	DRD1	1.6 (1.1-2.3)	0.64
	HTR1E	1.5 (1-2.2)	0.22
	HTR5A	0.51 (0.32-0.81)	0.45
	HTR3B	1.6 (1.1-2.3)	0.49
	GABRA1	1.5 (1-2.2)	0.49
	GABRA2	1.6 (1.1-2.3)	0.72
	GABRA3	0.57 (0.33-0.99)	0.32
	GABRA5	1.8 (1.2-2.6)	0.17
	GABRB2	1.5 (1.1-2.2)	0.47
	GABRD	1.9 (1.3-2.9)	0.094
	GABRG2	1.5 (1-2.3)	0.82
	GABRG3	1.5 (1-2.3)	0.075
	GRIA3	0.64 (0.39-1)	0.5
	GRIN1	1.4 (0.98-2.1)	0.11
	GRIN3A	1.4 (0.85-2.2)	0.46
	GRM5	1.6 (1.1-2.3)	0.73
	GRM7	1.5 (1.1-2.2)	0.26
	CHRM3	1.9 (1.1-3.2)	0.85
	CHRM4	2 (1.3-3.2)	0.2
	CHRNA5	1.8 (1.1-3.2)	0.79
	CHRNA7	0.69 (0.46-1)	0.93
	CHRNB2	1.8 (1.2-2.7)	0.95
	ADRA1B	1.7 (1.1-2.5)	0.37
	ADRA2A	1.7 (1.1-2.7)	0.77
	ADRA2C	1.9 (1.2-3.1)	0.2

TCGA cancer	Gene symbol	HR (95% CI)	p value
LGG	DRD1	0.49 (0.3-0.79)	0.68
	HTR1E	0.62 (0.4-0.96)	0.58
	HTR2A	0.41 (0.29-0.58)	0.069
	HTR5A	0.44 (0.28-0.7)	0.24
	HTR3B	0.51 (0.27-0.95)	0.43
	GABRA1	0.4 (0.27-0.59)	0.43
	GABRA2	1.7 (1-2.8)	0.77
	GABRA4	0.61 (0.43-0.87)	0.78
	GABRA5	0.6 (0.39-0.91)	0.28
	GABRB2	0.5 (0.32-0.77)	0.84
	GABRD	0.28 (0.19-0.4)	0.038*
	GABRG2	0.3 (0.2-0.47)	0.41
	GRIA4	0.35 (0.25-0.49)	0.00072***
	GRID2	0.41 (0.29-0.58)	0.81
	GRIK1	4.1 (2.8-6.1)	0.0071**
	GRIK4	0.28 (0.2-0.4)	0.27
	GRIN1	0.28 (0.18-0.44)	0.24
	GRIN2A	0.44 (0.27-0.73)	0.11
	GRIN2B	0.46 (0.31-0.68)	0.28
	GRM2	0.59 (0.42-0.84)	0.8
	GRM3	0.51 (0.25-1)	0.51
	GRM4	0.41 (0.28-0.61)	0.085
	GRM7	0.64 (0.45-0.93)	0.35
	CHRM1	0.49 (0.32-0.73)	0.52
	CHRM3	1.9 (1.3-2.9)	0.12
	CHRNA2	0.46 (0.29-0.72)	0.91
	CHRNB1	3 (1.9-4.8)	0.081
	ADRA1B	0.46 (0.32-0.66)	0.73
	ADRA2C	0.46 (0.3-0.7)	0.0046*

Table S3. KEGG enrichment analysis.

Pathway	Total	Expected	Hits	P.Value	FDR	Genes involved
GABAergic synapse	89	0.104	5	2.19E-08	6.95E-06	GABRA4, PRKCG, GABRA1, GABRB2,TRAK2
Morphine addiction	91	0.106	4	2.16E-06	0.000343	GABRA4, PRKCG, GABRA1, GABRB2
Nicotine addiction	40	0.0465	3	1.05E-05	0.00112	GABRA4, GABRA1, GABRB2
Retrograde endocannabinoid signaling	148	0.172	4	1.50E-05	0.0012	GABRA4, PRKCG, GABRA1, GABRB2
Serotonergic synapse	115	0.134	3	0.000252	0.016	APP, PRKCG, GABRB2
Taste transduction	83	0.0965	2	0.0039	0.201	GABRA4, GABRA1
Fc gamma R-mediated phagocytosis	91	0.106	2	0.00467	0.201	PRKCG, PRKCD
Inflammatory mediator regulation of TRP channels	100	0.116	2	0.00561	0.201	PRKCG, PRKCD
Neuroactive ligand-receptor interaction	338	0.393	3	0.0057	0.201	GABRA4, GABRA1, GABRB2
Vascular smooth muscle contraction	132	0.154	2	0.00961	0.306	PRKCG, PRKCD

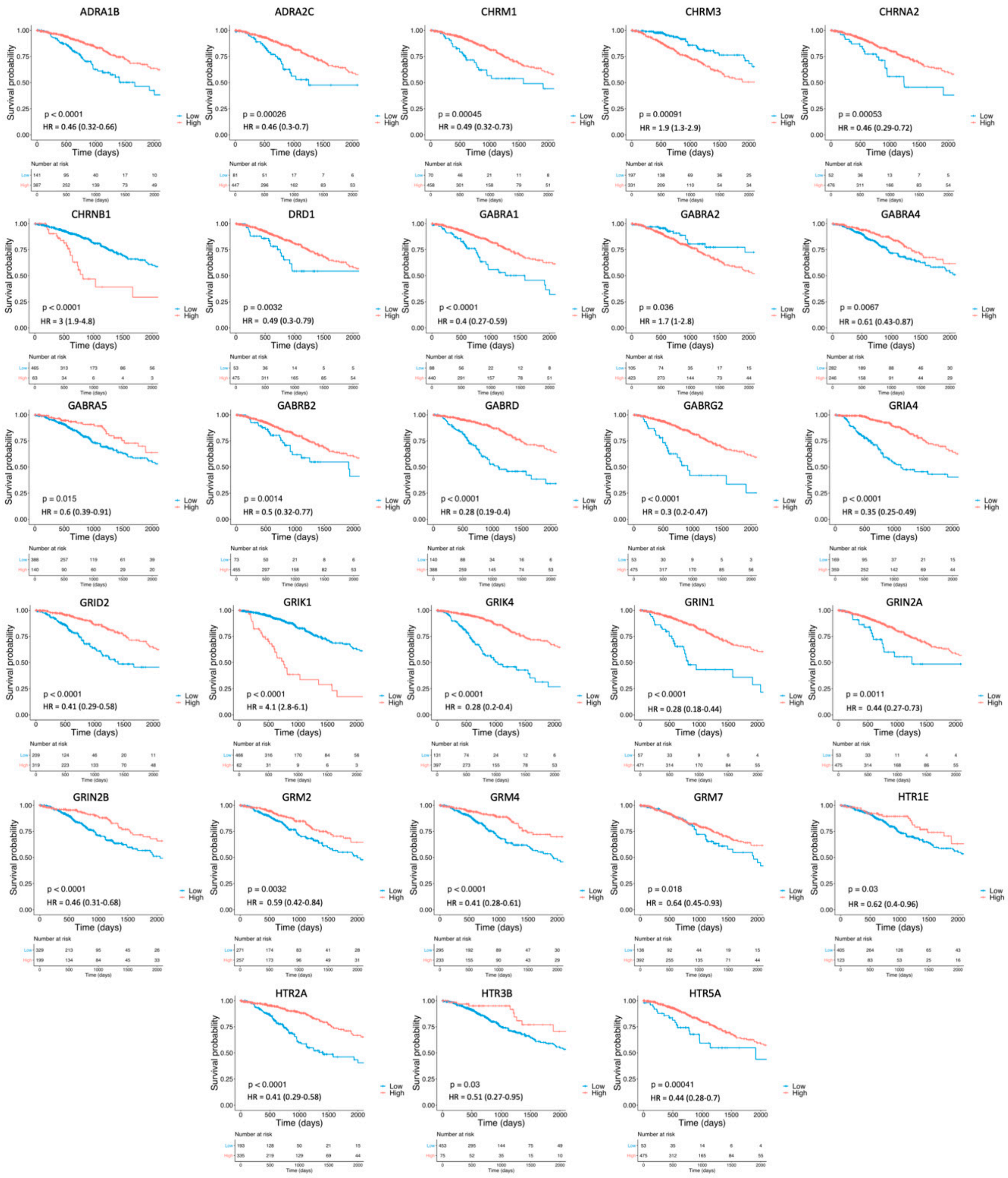

Figure S1. Overall survival probabilities as a function of the levels of the differentially expressed genes in LGG. Only the statistically significant (P≤ 0.05, log-rank test) cases are shown. The effect on overall survival probabilities of each NT receptor gene was assessed using a Cox Proportional-Hazard Model and the hazard ratio (HR) is shown.

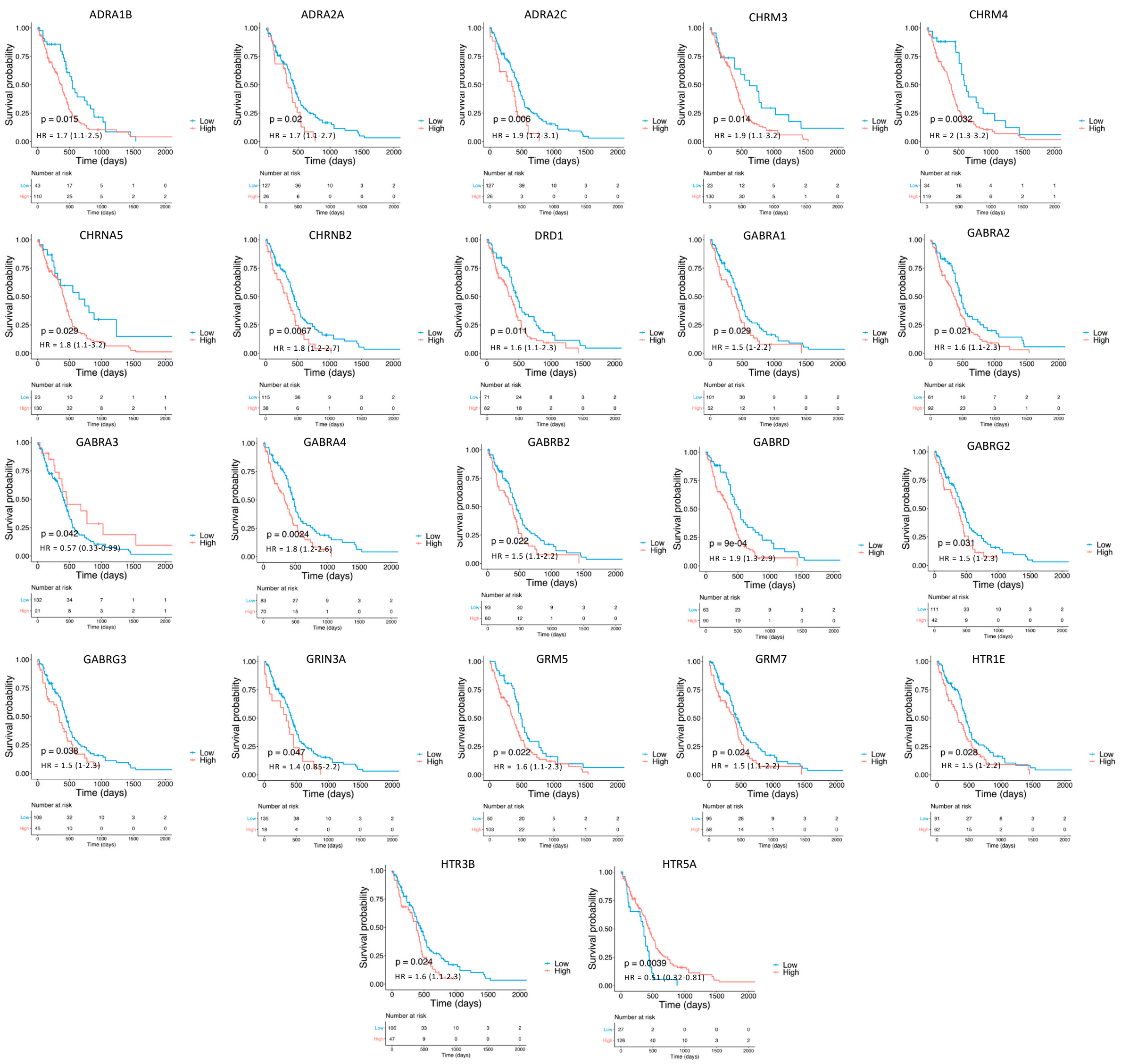

Figure S2. Overall survival probabilities as a function of the levels of the differentially expressed genes in GBM. Only the statistically significant (P≤0.05, log-rank test) cases are shown. The effect on overall survival probabilities of each NT receptor gene was assessed using a Cox Proportional-Hazard Model and the hazard ratio (HR) is shown.

A LGG - TCGA

	beta	HR (95% CI for HR)	Wald test	p value
**Risk group**	-1	0.35 (0.22-0.55)	21	5.40E-06
**Age**	0.056	1.1 (1-1.1)	63	1.70E-15
**Gender**	-0.13	0.88 (0.62-1.2)	0.56	0.45

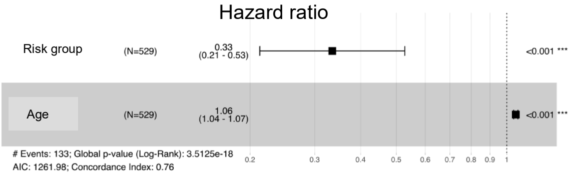

B LGG - GSE107850

	beta	HR (95% CI for HR)	Wald test	p value
**Risk group**	-0.8	0.45 (0.28-0.71)	12	0.00061
**Age**	-0.028	0.97 (0.95-0.99)	8.4	0.0037
**Gender**	-0.31	0.74 (0.49-1.1)	2.3	0.13

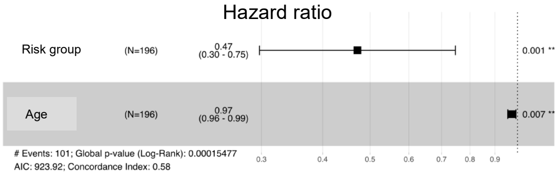

C GBM - TCGA

	beta	HR (95% CI for HR)	Wald test	p value
**Risk group**	0.54	1.7 (1.2-2.5)	7.8	0.0051
**Age**	0.025	1 (1-1)	12	0.00061
**Gender**	0.084	1.1 (0.75-1.6)	0.19	0.66

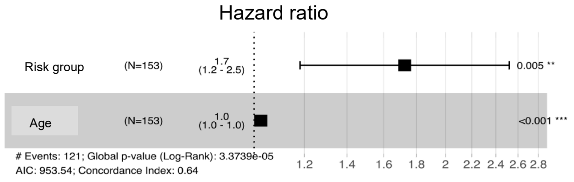

D GBM - GSE4412

	beta	HR (95% CI for HR)	Wald test	p value
**Risk group**	1.1	2.9 (1.6-5.3)	12	0.00057
**Age**	0.0078	1 (0.99-1)	0.58	0.45
**Gender**	0.072	1.1 (0.6-1.9)	0.06	0.81

Figure S3. External validation of the prognostic value of the gene signatures identified in the TCGA brain cancers. (A) Outcomes of the univariate and multivariate analysis of the neurotransmitters-associated prognostic index for low grade glioma using the data from TCGA. (B) Validation of the prognostic index in (A) on an external dataset (accession code GSE107850). (C) Outcomes of the univariate and multivariate analysis of the neurotransmitters-associated prognostic index for glioblastoma multiforme (GBM) the data from TCGA. (D) Validation of the prognostic index in (C) on an external dataset (accession code GSE4412-GPL96). The prognostic factor "risk group" was compared with other clinical confounders using univariate analysis using the Cox proportional-hazard model, as shown in tables. The forest plots show the outcome of the multivariate analysis of the factors that were statistically significant at the univariate analysis.

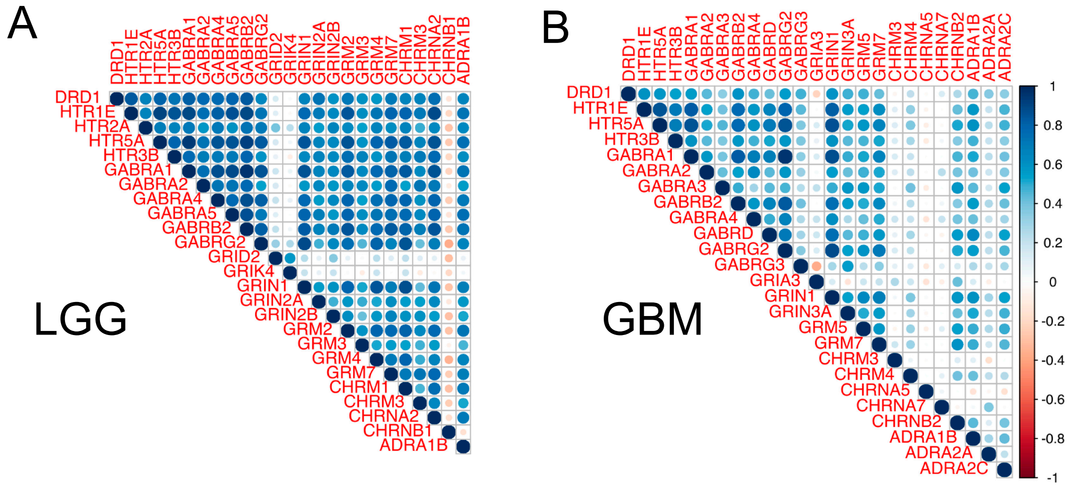

Figure S4. Correlation among differentially expressed prognostic NTR genes in brain cancers. (A) Low-grade glioma (LGG). (B) Glioblastoma multiforme (GBM). Colour intensity and the size of the dots are proportional to the Pearson correlation coefficients.

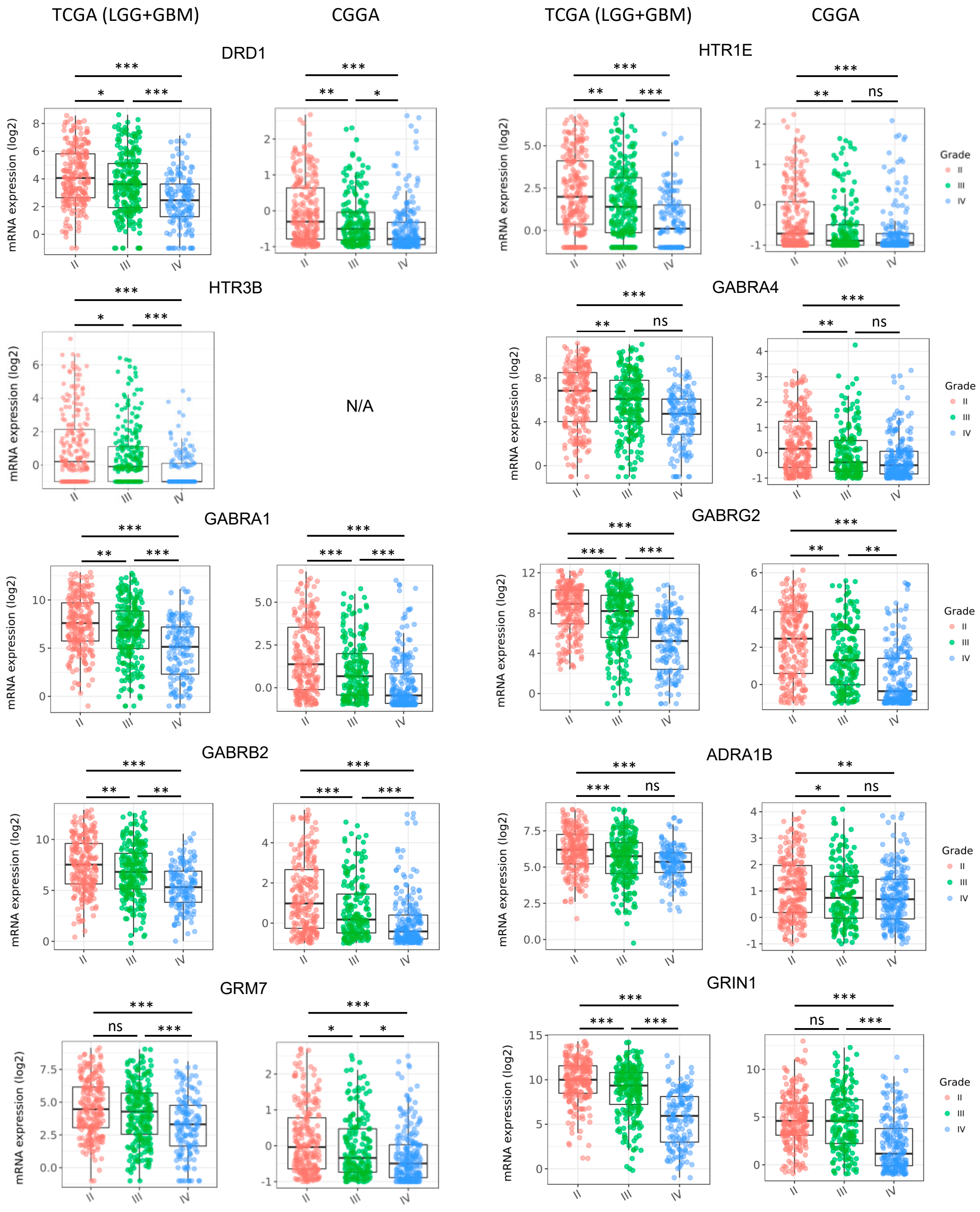

Figure S5. Gene expression levels of the 10-NTR genes as a function of the cancer grade. Both TCGA (The Cancer Genome Atlas) and CGGA (Chinese Glioma Genome Atlas) datasets were accessed. The data were classified based on the WHO grade. The centre line indicates the median. The boxes show the distance between the first and third quartile with the whiskers extending up to 1.5 times the interquartile range, the line is the median. The Tukey's Honest Significant Difference (HSD) was used to test estimate the difference in expression levels among the different grades. *** $p \leq 0.001$; ** $p \leq 0.01$; * $p \leq 0.05$; ns, not significant. The gene HTR3B was not available in the CGGA dataset.

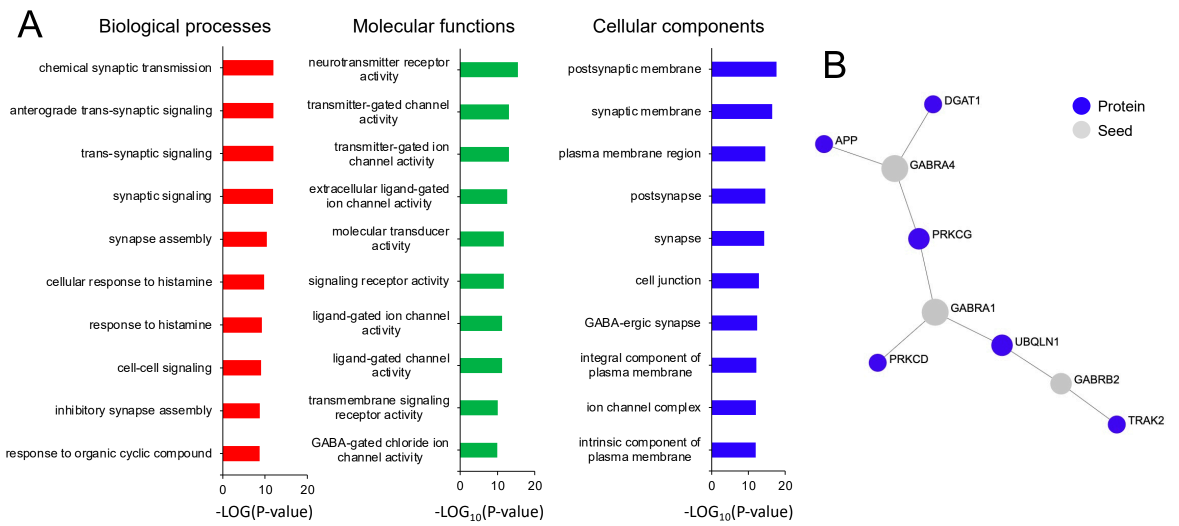

Figure S6. Gene ontology enrichment and network analysis of the 10-NTR genes. (A) Top 10 enrichment scores in the Gene Ontology (GO) enrichment analysis of the NTR 10 genes with opposite association with clinical outcomes between LGG and GBM: DRD1, HTR1E, HTR3B, GABRA1, GABRA4, GABRB2, GABRG2, GRIN1, GRM7, and ADRA1B. (B) Brain-specific protein-protein interactions (PPI) network analysis. The subnetwork contains 9 nodes, 8 edges and 3 seeds.

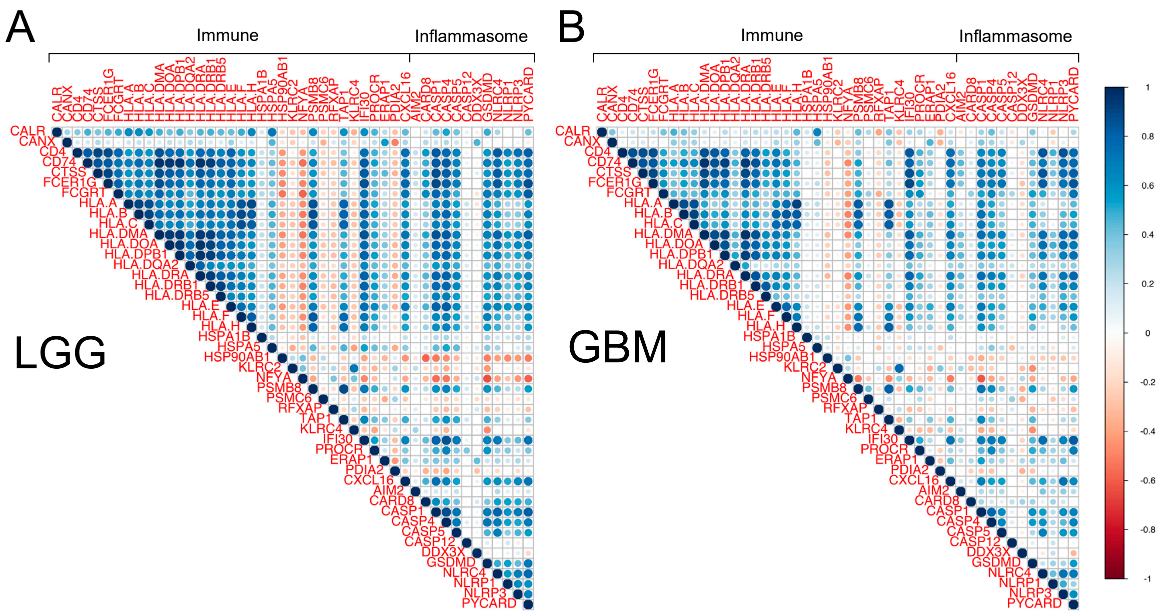

Figure S7. Correlation between immune and inflammasome gene panels in brain cancers. (A) Low-grade glioma (LGG). (B) Glioblastoma multiforme (GBM). Colour intensity and the size of the dots are proportional to the Pearson correlation coefficients.

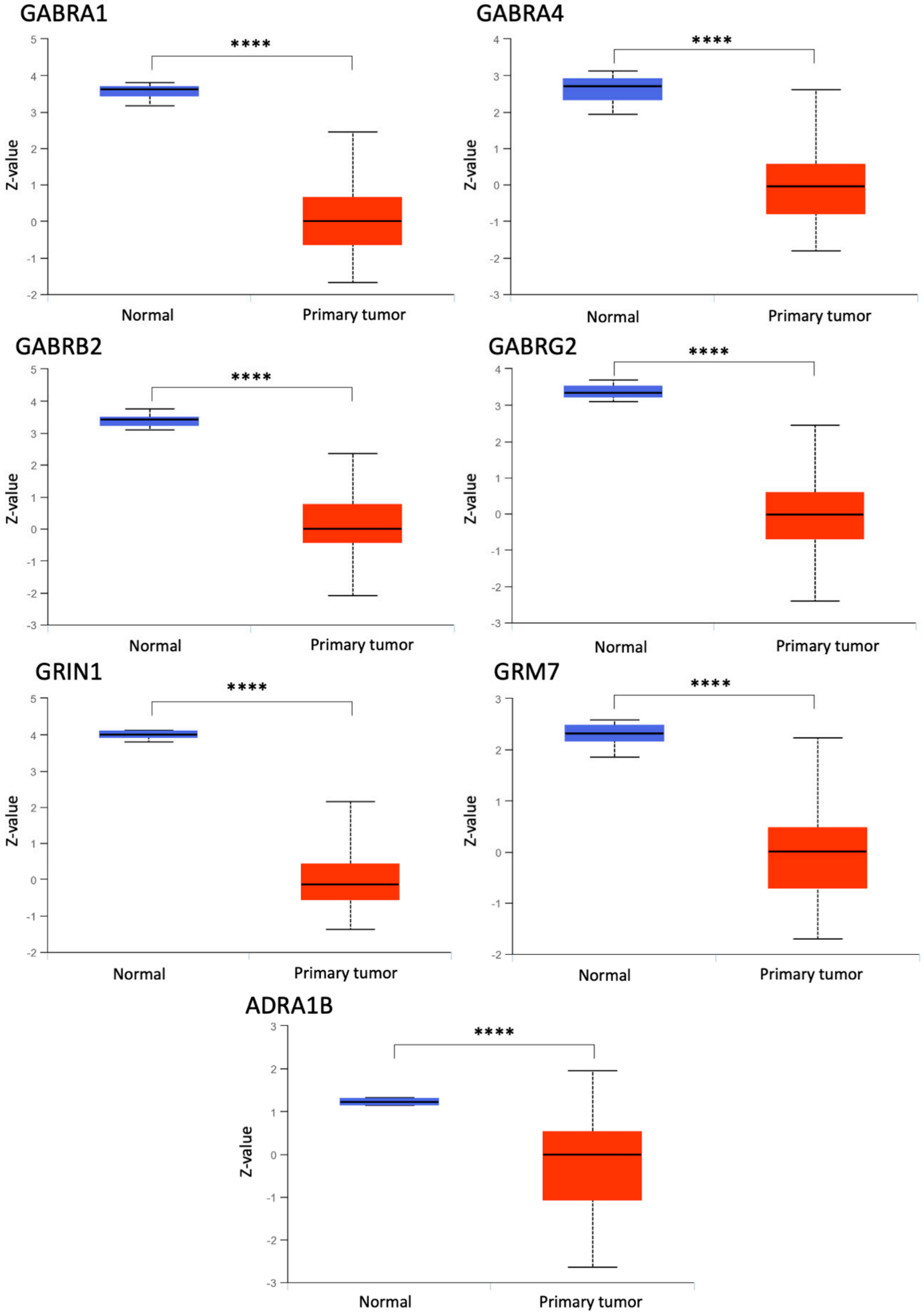

Figure S8. Protein expression levels of the NTR genes in GBM and normal tissue. Seven out of the ten proteins encoded by the NTR genes were found. Z-values show the standard deviations from the median across samples for each type of tissue. The spectral count ratio values from the Clinical Proteomic Tumor Analysis Consortium (CPTAC) were first log2 transformed, normalized within each sample profile, and then normalized across samples. The University of ALabama at Birmingham CANcer data analysis Portal (UALCAN) was used to visualize the box plots and compute the two-sided t-test between the two groups. **** $p \leq 0.0001$.
